# Modeling Test-Taking Non-effort in MIRT Models

**DOI:** 10.3389/fpsyg.2019.00145

**Published:** 2019-02-04

**Authors:** Yue Liu, Zhen Li, Hongyun Liu, Fang Luo

**Affiliations:** ^1^Beijing Key Laboratory of Applied Experimental Psychology, Faculty of Psychology, Beijing Normal University, Beijing, China; ^2^eMetric LLC, San Antonio, TX, United States

**Keywords:** test-taking effort, multidimensional item response theory, effort-moderated model, missing data, response time

## Abstract

The validity of inferences based on test scores will be threatened when examinees' test-taking non-effort is ignored. A possible solution is to add test-taking effort indicators in the measurement model after the non-effortful responses are flagged. As a new application of the multidimensional item response theory (MIRT) model for non-ignorable missing responses, this article proposed a MIRT method to account for non-effortful responses. Two simulation studies were conducted to examine the impact of non-effortful responses on item and latent ability parameter estimates, and to evaluate the performance of the MIRT method, comparing to the three-parameter logistic (3PL) model as well as the effort-moderated model. Results showed that: (a) as the percentage of non-effortful responses increased, the unidimensional 3PL model yielded poorer parameter estimates; (b) the MIRT model could obtain as accurate item parameter estimates as the effort-moderated model; (c) the MIRT model provided the most accurate ability parameter estimates when the correlation between test-taking effort and ability was high. A real data analysis was also conducted for illustration. The limitation and future research were discussed further.

Test validity is at risk when examinees are not fully engaged during testing. Test-taking effort, typically defined as a student's engagement and expenditure of energy toward the goal of attaining the highest possible score on the test (Debeer et al., 2014), has been a growing concern in psychological and educational measurement. Wise and Kong (2005) noted three situations where non-effortful responses could happen: (a) assessment programs (e.g., PISA) that have serious potential consequences for institutions but few consequences for examinees; (b) high-stakes testing programs that sometimes administer test items in low-stakes settings, such as in the pilot study of a test program (Cheng et al., 2014); (c) a substantial amount of measurement studies conducted in low-stakes settings at colleges and universities. Additionally, the non-effortful behavior can also manifest in high-stakes contests. For example, Bridgeman and Cline (2004) found that about half the examinees were forced to guess on the last six items to finish the CAT-GRE analytical section before time expired.

Previous studies have shown that non-effortful responses put into question the validity of score-based inferences by weakening the connection between scores and examinees' true abilities (Wise and DeMars, 2006, 2010; Wise, 2015; Weirich et al., 2016). First, when an unidimensional item response theory (IRT) model is applied to test scoring, test-taking non-effort leads to biased estimations of both item parameters and latent abilities (Wise and DeMars, 2006). Due to the biased estimation of discrimination parameters, test information, and standard errors of measurement can also be biased (Wise and DeMars, 2006). Next, the measured construct could be different from the theoretically tested construct and a decrease in convergent validity may occur as well (Wise and DeMars, 2006; Weirich et al., 2016). Furthermore, as test-taking non-effort usually occurs in low-stakes assessments, whose purpose is evaluating the group-level achievements, the impact of non-effortful responses on aggregated scores has recently been investigated. It was shown that the group means would be underestimated by around 0.20 SDs if the total amount of non-effortful responses exceeded 6.25, 12.5, and 12.5% for easy, moderately difficult, and difficult tests respectively (Rios et al., 2017).

In the awareness of test-taking non-effort's threat to measurement properties, researchers have recommended several approaches to deal with the non-effortful responses at different stages of testing. A basic one is to enhance the examinees' test-taking motivation, for instance, through making the test outcomes part of a grading system to increase the stakes of the assessment or by explaining the importance of the assessments to the examinees (Wise and DeMars, 2005; Liu et al., 2012). Another approach is effort filtering. After response data are collected, non-effortful responses are flagged and deleted from the original data (Sundre and Wise, 2003; Wise and DeMars, 2005). Results from several studies have shown that removing the non-effortful responses can increase the average test performance (Wise et al., 2006; Wise and DeMars, 2010; Swerdzewski et al., 2011; Steedle, 2014). However, the approach is based on an assumption that the test-taking effort and the actual proficiency level are unrelated, which might be violated in real situations. A third approach to addressing the low-effort issue is to include test-taking effort in the measurement model, named effort models, which has been shown to be the most effective and flexible (Wise and DeMars, 2006). We summarized the existed effort models as follows.

## Effort Models

Psychometric models accounting for test speededness or motivation changes during testing have been continuously proposed and studied for decades (Yamamoto and Everson, 1995; Wise, 1996; Cao and Stokes, 2008; Goegebeur et al., 2008; Meyer, 2010; Jin and Wang, 2014; Mittelhaëuser et al., 2015). A few of them are also suitable for dealing with non-effortful responses. The most common type is what we will refer to as switching models, including the absorbing state models and the gradually decreasing effort models. In the absorbing state models (e.g., the HYBRID model), it is assumed that all test-takers begin with an effortful state, but during the test some switch to a non-effortful state suddenly and begin to give random responses (Yamamoto and Everson, 1995; Wise, 1996; Jin and Wang, 2014). Similarly, the gradually decreasing effort models also assume an equally effortful state at the beginning, but instead of a sudden switch to random responding, some test-takers begin exhibiting gradually decreasing effort (Cao and Stokes, 2008; Goegebeur et al., 2008). The switching models have a strong assumption that once examinees switch to non-effortful behaviors, they won't switch back to effortful behaviors. However, this assumption might be violated in practice. For example, Wise and Kong (2005) discovered that the non-effort behaviors occurred throughout the test, and not just toward the end. Therefore, a model which allows for switching back and forth between non-effortful and effortful behavior on different items will be preferred. Mixture models have also been applied to account for effortful and non-effortful groups by imposing constraints on item difficulties or average response times (Meyer, 2010; Mittelhaëuser et al., 2015). This type of method has been criticized in two aspects. On one hand, the assumption of the parameter relationships between two classes may not hold in practice (Mittelhaëuser et al., 2015). On the other hand, the model simply divides students into two classes, ignoring the fact that everyone may become low-effortful at some point during the test. The third type of model is named the effort-moderated model, which is supposed to adequately represent how test-takers behave in a real test (Wise and DeMars, 2006). In the effort-moderated model, two different item response functions are specified—one for effortful behaviors and the other for non-effortful behaviors. Due to its simplicity and flexibility, this model has been increasingly studied and used to report scores in educational tests. Apart from establishing time thresholds, it does not require additional parameter estimation (Wise and Kingsbury, 2016). Moreover, it allows non-effortful response to occur at any point of the test and does not require assumptions about the patterns of non-effort behaviors. However, some practical limitations exist in the application of the effort-moderated model. For example, when there is a large proportion of non-effortful responses (i.e., above 80%), this method fails due to unacceptably large standard errors of scores (Wise and Kingsbury, 2016).

In summary, in spite of decades of research efforts, it is still unclear when and how non-effortful responses should be dealt with in practice. Quite a few research questions remain to be answered. For example, is it possible to obtain parameter estimates as accurate as the effort-moderated model by using a MIRT model? Can we estimate examinees' ability and propensity of giving effortful responses simultaneously? What's the relationship between these two latent traits? Therefore, this article has two objectives: (a) to evaluate the degree of non-effortful responses' impact on parameter estimates in various simulated conditions; (b) to apply the multidimensional item response theory (MIRT) models for handling non-ignorable missing responses to deal with non-effortful responses and evaluate its performance. Two simulation studies were conducted to compare the MIRT model with the unidimensional 3PL model (denoted as 3PL model) and the effort-moderated model in various conditions. Specifically, for the first objective, the 3PL model was applied as a baseline to assess the impact of non-effortful responses on parameter estimates in all simulation conditions. For the second objective, the performance of the MIRT model was evaluated in two simulation studies. Study I generated data based on the effort-moderated model, as a previous research did (Rios et al., 2017), while Study II generated data based on the MIRT model for comparison. As the generating model for non-effortful responses were different in the two simulation studies, results from these studies have the potential to inform practitioners of: (1) the conditions in which non-effortful responding is a major concern for parameter estimation; (2) whether the MIRT method is as valid and effective as the effort-moderated model for purifying biased estimates regardless of different possible mechanism or causes of non-effortful responses. In addition, the three models were applied to a real data set for illustration.

In the following sections, the effort-moderated model and the proposed MIRT model are described in details. Then the design, procedure, and results of Study I and Study II are illustrated, followed by a real data illustration. The theoretical and practical inferences from the studies, limitation and future research are discussed at the end.

## Models of Handling Non-effortful Responses

### The Effort-Moderated Model

In the effort-moderated model, test-takers' responses are assumed to be generated by either rapid-guessing behaviors or solution behaviors (Wise and DeMars, 2006). Under rapid-guessing behaviors, for multiple choice (MC) items, the probability of a correct response to an item is a constant value at (or near) the chance level regardless of the test taker's achievement level. In contrast, under solution behaviors, the probability of a correct response to an item increases with test-takers' achievement levels and can be effectively modeled with a monotonically increasing function such as a unidimensional IRT model. Wise and DeMars (2006) used response time to flag rapid-guessing behaviors and built the effort-moderated model as follows.

Suppose that for item *j*, there is a time threshold *T*_*j*_ that differentiates a rapid-guessing behavior from a solution behavior. Given a test taker *i*'s response time on item *j, RT*_*ij*_, a dichotomous index of solution behavior (effortful response) *F*_*ij*_, can be computed by comparing *RT*_*ij*_ to *T*_*j*_. If the solution behavior is represented by the three-parameter logistic (3PL) model, and the rapid-guessing behavior is represented by a constant probability model specified as $P_{j}\left(\theta_{i}\right)=\frac{1}{h_{j}}$, where *h*_*j*_ is the number of options for item *j*, the effort-moderated model would be

(1) $$\begin{array}{l} P_{j}\left(\theta_{i}\right)=\left(F_{ij}\right)\left(c_{j}+\left(1-c_{j}\right)\left(\frac{e^{a_{j}\left(\theta_{i}-b_{j}\right)}}{1+e^{a_{j}\left(\theta_{i}-b_{j}\right)}}\right)\right)+\left(1-F_{ij}\right)\left(\frac{1}{h_{j}}\right) \end{array}$$

(2) $$\begin{array}{l} F_{ij}=\{\begin{array}{c} 1,if\;RT_{ij}\geq T_{j}\\ 0,otherwise \end{array} \end{array}$$

where *P*_*j*_(θ_*i*_) is the probability of a correct response to an item *j* of examinee *i, a*_*j*_, *b*_*j*_, *c*_*j*_ indicate the discrimination parameter, difficulty parameter, and guessing parameter for item *j* respectively.

As the probability of passing an item under non-effortful responding is assumed to be the same regardless of the test-takers' achievement levels, it is equal to adding a constant to the likelihood function. Because the constant probability do not influence where the likelihood function for an item or examinee peaks, there is no need to consider the value of the constant probability ($\frac{1}{h_{j}}$) when estimating parameters. In other words, test-scoring using the effort-moderated model is equivalent to filtering out non-effortful responses when calculating the likelihood functions. As the effort-moderated achievement estimates for rapid guessers are based on reduced numbers of item responses, their standard errors are higher than those associated with achievement estimates from test-takers who exhibited solution behavior to all the items (Wise and Kingsbury, 2016).

### The MIRT Model for Non-effortful Responses

The MIRT model that accounts for non-ignorable missing responses has been well studied (Rose et al., 2010; Rose, 2013). It typically assumes that a latent response propensity variable, represented by the missing indicator variables, predicts the propensity to omit an item. Similarly, we suppose that a latent variable underlying the dichotomous index of effortful response *F*_*ij*_ from the effort-moderated model, which represented the propensity of effortful responses, can be regarded as the latent response propensity variable as in the MIRT model for missing responses. Subsequently, the joint model of item responses and the index of effortful responses can be applied to estimate the latent ability and the latent effortful propensity simultaneously. In this article, the between-item MIRT model was chosen as the representative of MIRT models for non-effortful responses (Rose et al., 2016). For an extensive introduction to the MIRT model, please refer to a book by Reckase (2009).

**Figure 1:**
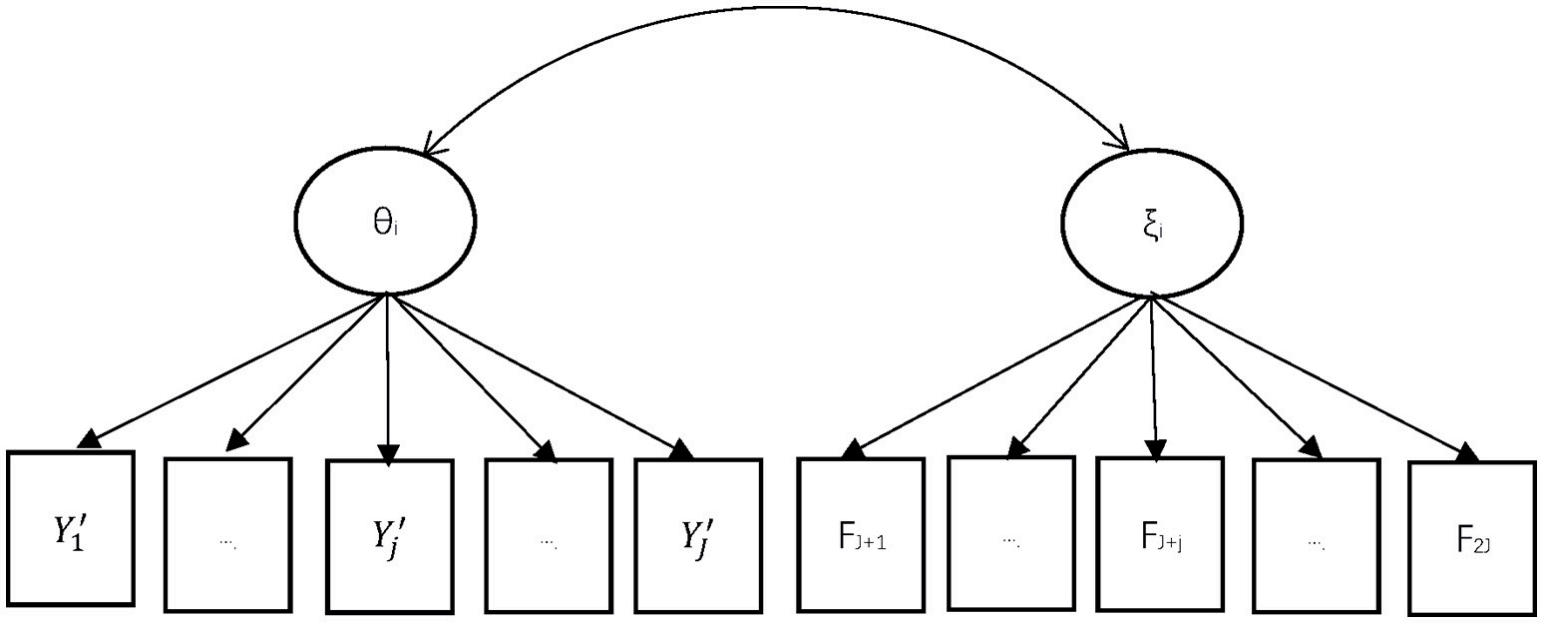
MIRT Approach for Non-effortful Responses. *Y*_*j*_' is the response for item *j* after non-effortful responses were recoded as missing values, where *Y*_*j*_' = 1 for correct effortful response, *Y*_*j*_' = 0 for incorrect effortful response, *Y*_*j*_' = missing for non-effortful responses *(j* = 1,…,*J). F*_*J*+*j*_ is the indicator for effortful response for item *j*, where *F*_*J*+*j*_ = 1 indicates effortful response, *F*_*J*+*j*_ = 0 indicates non-effortful response *(j* = 1,…,*J)*. θ_*i*_ is the latent variable for the ability of person *i (i* = **1,…**,*N)*. ξ_*i*_ is the latent variable for the propensity to response effortfully of person *i* (*i* = 1,…,N).

Figure 1 depicted an example of the proposed MIRT model. Suppose the response on the test item *j* is denoted by *Y*_*j*_, while *Y*_*j*_′ represented the response with any non-effortful response recoded as missing value. The manifest effortful response indicator is represented by *F*_*j*_. Therefore, the original dataset should be reorganized by matching these two parts (*Y*_*j*_′ *F*_*j*__)_ for each examinee. Two latent variables are estimated within the MIRT framework: the latent ability θ and the latent effortful propensity ξ. They are assumed to follow a bivariate normal distribution. Under the assumption of local stochastic independence, all the manifest variables given the latent variables θ and ξ in the model should be independent.

The effortful response indicators (*F*_*ij*_) can be predicted by ξ using any unidimensional IRT model, such as the Rasch model and Birnbaum's two- or three-parameter model (Rose et al., 2016). Holman and Glas (2005) introduced their MIRT model using a two-parameter logistic (2PL) model for both latent ability and missing propensity. But they noted that a 1PL model may be more convenient for the measurement model of the missing indicators. In the current study, the Rasch model is chosen as the measurement model for the effortful behavior indicators, while the 3PL model is chosen for the effortful responses. Therefore, in the framework of the between-item multidimensional model, the MIRT model for non-effortful responses would be:

(3) $$\begin{array}{l} P\left(U_{ik}=1|\delta_{\text{i}}\;,\;{\text{a}}_{k},\;h_{k},\;c_{k},\;d_{k}\right)=\left(F_{ik}\right)\\ \left(c_{k}+\left(1-c_{k}\right)\frac{e^{{\text{a}}_{k}\delta_{\text{i}}´+d_{k}}}{1+e^{{\text{a}}_{k}´\delta_{i}+d_{k}}}\right)+\left(1-F_{ik}\right)\left(\frac{1}{h_{k}}\right) \end{array}$$

where *U* = (*Y', F*), and *J* is the number of items. ´*Y*_*ik*_ is the recoded responses, and *F*_*ik*_ is the effortful response indicator variable for *J* items.

For 1 < *k* ≤ *J:*

(4) $$\begin{array}{l} \overset{´}{Y_{ik}}=\{\begin{array}{c} 1,\;correct\;effortful\;response\\ \;0,\;incorrect\;effortful\;reponse\\ missing,\;non-effortful\;response \end{array} \end{array}$$

For *J*<*k* ≤ *2J:*

(5) $$\begin{array}{l} F_{ik}=\{\begin{array}{c} 1,\;effortful\;response\\ 0,non-effortful\;response \end{array} \end{array}$$

***a***_***k***_ is a vector of item discrimination parameters, where for 1 ≤ *k* ≤ *J*, ${\vec{a}}_{k}$ = (*a*_*k*_, 0), and fo*r J*<*k* ≤ *2J*, $a_{k}=\left(0,1\right)$. *d*_*k*_ is the intercept for MIRT model (for 1 ≤ *k* ≤ *J*,$b_{k}=\frac{-d_{k}}{\sqrt{\overset{⇀}{a_{k}}\overset{´}{\overset{⇀}{a_{k}}}}}$, where *b*_*k*_ is the difficulty parameter; for *J*<*k* ≤ *2J*, *b*_*k*_ = −*d*_*k*_, where *b*_*k*_ means the difficulty parameter in the Rasch model for effortful behavior indicators). *C*_*k*_ is the guessing parameter (for, *J*<*k* ≤ 2*J, C*_*k*_ = 0). The ability vector of each examinee is **δ**_**i**_ = (θ_*i*_, ξ_*i*_).

## Study I

### Design

#### Data Generation

Response data were generated based on the effort-moderated model [Equation (1 and 2)] for a 60-item test with two types of responses: (1) effortful responses and (2) non-effortful responses. The test only contains 4-option MC items. For effortful responses, data were generated based on the standard 3PL model. For non-effortful responses, which is defined as rapid guesses in this study, the probability of a correct response equals to chance: *P*_*i*_(θ) = 0.25 (Wise and DeMars, 2006). At individual level, simulees providing all effortful responses were labeled as effortful simulees, while those providing at least one non-effortful responses were categorized as non-effortful simulees. Latent abilities (θ_*i*_) for 2,000 simulees were randomly sampled from *N* (0, 1^2^), while true item parameters for effortful responses were generated by the following distributions:

(6) $$\begin{array}{l} a_{j}\sim N\left(0.8,\;0.2^{2}\right)\\ b_{j}\sim N\;\left(\bar{b_{j}},1^{2}\right)\\ c_{j}=\frac{1}{h_{j}}=0.25 \end{array}$$

where *b_j_* varied across conditions, and the pseudo-guessing parameter *c*_*j*_ was set to 0.25 for four-option MC items (Han, 2012).

#### Independent Variables

Four independent variables were manipulated in this study: (1) the percentage of non-effortful simulees in the sample (κ), (2) the percentage of non-effortful responses within a non-effortful simulee (π), (3) the correlation between non-effortful responding and ability (γ), (4) test difficulty (β).

Three different percentages of non-effortful simulees were manipulated (10, 25, and 50%), while three within-simulee levels of non-effortful responding were manipulated (10, 25, and 50%) and equally constrained for each of the non-effortful simulees. In combining the different levels of the two independent variables, we produced overall percentages of non-effortful responses (ρ) that have been seen in operation and previous studies: 1, 2.5, 5, 6.25, 12.5, and 25% (Wise and DeMars, 2006; Rios et al., 2017).

In addition, the correlation between effortful responding and latent ability also has three levels (0.0, 0.4, and 0.8). Positive correlations were employed based on the hypothesis that low ability examinees may be more prone to non-effortful responses, according to recent findings in social psychology and in psychometrics (Jagacinski and Nicholls, 1990; Thompson et al., 1995; Penk and Schipolowski, 2015; Rios et al., 2017). Therefore, in this current study, test-taking non-effort was assumed to be related to low ability as Rios et al. (2017) simulated in their study. The levels of 0.4 and 0.8 represented the medium and high correlations respectively. In contrast, the correlation level of 0.0 was set as a baseline.

The last independent variable was test difficulty. When generating item difficulty parameters, the mean varies at three levels: *β* = *b_j_* = −1, 0, and 1. Correspondingly, the three levels of test difficulties are: easy, *b*_*j*_~N (−1, 1^2^), moderately difficult, *b*_*j*_~*N* (0, 1^2^), and difficult, *b*_*j*_~ *N* (1, 1^2^).

The four independent variables and their respective levels were fully crossed (3 × 3 × 3 × 3 = 81 conditions in total). One hundred replications were simulated for each condition. Every generated dataset was analyzed by three models: (1) the 3PL model based on the original data, (2) the effort-moderated model based on the data with non-effortful responses flagged, (3) the MIRT model based on the reorganized data to estimate the latent ability and propensity to respond effortfully simultaneously.

### Estimation

Bock-Aitkin EM Algorithm was applied for estimating item parameters and expected a posteriori (EAP) approach was applied for estimating ability parameters by flexMIRT^®;^ (Cai, 2015). The guessing parameters under the 3PL model were estimated, with the prior distribution *logit*(*c*_*j*_)~*N*(−1.09, 0.5) for items whose *c* = 0.25 when generated (Cai, 2015). The c-parameters under the effort-moderated model were set at 0.25 to control for the standard errors of the estimates, as a previous study did (Wise and DeMars, 2006). Because the MIRT model has a similar measurement model as the effort-moderated model, its c-parameters were also constrained to be 0.25. As the primary goal of this study is to take non-effort into account in the models, not to detect non-effortful responses, the indicators of effortful responses (*F*_*ij*_) applied in these models were set as the true values for calibration. In this study, the data applied to the 3PL model contains responses with non-effortful responses (*Y*), the data used for the effort-moderated model is the response data with non-effortful responses recoded as missing values (*Y*′), the data applied to the MIRT model is a combination of (*Y*′) and effortful indicators (*F*). The true parameters are based on the responses without non-effort (Y^*^) in the framework of 3PL model, which is also the original generated data assuming all simulees responded regularly. As neither the data nor the model are the same, the models' parameter estimates might not always be based on the metric of the generating scale, which means that they are not comparable. Therefore, the scales of all the estimated parameters were transformed onto the scale of generating parameters by the Stocking-Lord's (SL) method (Kim and Cohen, 2002) after the calibration to compare with their true values. An R package called “plink” (Weeks, 2010) was used for linking.

### Evaluation Measures

To investigate the accuracy of item and ability parameter estimations of different methods, *BIAS, Root Mean Squared Error (RMSE)* and *correlation* for the parameters were analyzed across conditions.

(7) $$\begin{array}{l} BIAS=\frac{1}{R}\sum_{r=1}^{R}\frac{1}{T}\sum_{t=1}^{T}\left(\omega_{t}-{\hat{\omega }}_{t}\right) \end{array}$$

(8) $$\begin{array}{l} RMSE=\frac{1}{R}\sum_{r=1}^{R}\sqrt{\frac{1}{T}\sum_{t=1}^{T}{\left(\omega_{t}-{\hat{\omega }}_{t}\right)}^{2}} \end{array}$$

(9) $$\begin{array}{l} correlation=\frac{1}{R}\sum_{r=1}^{R}cor\left(\omega ,\hat{\omega }\right) \end{array}$$

where ${\hat{\omega }}_{t}$ denoted the parameter estimate, and ω_*t*_ denoted the true value. For item parameters, *T* denoted the number of items. For ability parameters, *T* denoted the number of examinees. *R* denoted the number of replications under each condition. The *correlation* was only computed for ability estimates and averaged using Fisher Z-r transformation.

## Results

### Recovery of Parameter Estimates

Table 1 presents the *BIAS* and *RMSE* of item parameter estimates for different models across various conditions. In general, for all of the conditions considered in this study, the *RMSE* of parameter estimates by the MIRT model or the effort-moderated model were much smaller than the 3PL estimates and barely any difference between the estimates of item parameters under the former two models can be observed.

**Table 1:** BIAS and RMSE of item parameter estimates under different models in study I.

**Measures**	**Condition**	**Level**	**κ**	**π**	**a**			**b**			**c**
					**3PL**	**Moderated**	**MIRT**	**3PL**	**Moderated**	**MIRT**	**3PL**
*BIAS*	ρ	1%	10%	10%	−0.019	−0.008	−0.009	−0.016	0.000	0.002	−0.002
		2.50%	10%	25%	0.021	−0.009	−0.009	0.047	0.001	0.003	0.013
			25%	10%	−0.005	−0.008	−0.009	−0.008	−0.002	0.000	0.000
		5%	10%	50%	0.119	−0.009	−0.009	0.302	0.003	0.005	0.066
			50%	10%	0.002	−0.009	−0.009	−0.013	−0.002	−0.003	−0.002
		6.25%	25%	25%	0.051	−0.009	−0.009	0.072	−0.001	0.001	0.019
		12.50%	25%	50%	0.141	−0.010	−0.010	0.331	0.002	0.004	0.072
			50%	25%	0.004	−0.010	−0.010	−0.073	−0.001	−0.001	−0.013
		25%	50%	50%	0.108	−0.011	−0.011	0.201	0.000	0.001	0.044
	γ	0.0			0.025	−0.009	−0.009	0.042	−0.001	0.000	0.011
		0.4			0.049	−0.009	−0.009	0.098	0.001	0.001	0.022
		0.8			0.067	−0.009	−0.010	0.142	0.000	0.004	0.032
	β	−1			0.011	−0.008	−0.008	−0.008	0.013	0.014	0.002
		0			0.044	−0.009	−0.009	0.104	−0.001	0.001	0.023
		1			0.086	−0.011	−0.012	0.185	−0.012	−0.011	0.040
*RMSE*	ρ	1%	10%	10%	0.146	0.101	0.101	0.217	0.173	0.174	0.039
		2.50%	10%	25%	0.137	0.102	0.102	0.236	0.177	0.177	0.043
			25%	10%	0.144	0.100	0.101	0.227	0.179	0.178	0.040
		5%	10%	50%	0.176	0.102	0.102	0.385	0.178	0.178	0.074
			50%	10%	0.146	0.104	0.103	0.250	0.182	0.181	0.043
		6.25%	25%	25%	0.148	0.105	0.105	0.284	0.188	0.187	0.051
		12.50%	25%	50%	0.210	0.109	0.110	0.434	0.197	0.196	0.083
			50%	25%	0.189	0.109	0.108	0.410	0.195	0.193	0.074
		25%	50%	50%	0.227	0.117	0.116	0.442	0.214	0.212	0.077
	γ	0.0			0.170	0.105	0.105	0.297	0.185	0.185	0.053
		0.4			0.165	0.104	0.104	0.312	0.183	0.182	0.057
		0.8			0.172	0.107	0.108	0.353	0.193	0.191	0.064
	β	−1			0.148	0.096	0.096	0.339	0.206	0.206	0.057
		0			0.155	0.101	0.101	0.288	0.157	0.157	0.056
		1			0.204	0.119	0.120	0.335	0.197	0.196	0.061

*3PL represents the standard 3PL model, moderated represents the effort–moderated model, MIRT represents the MIRT model*.

The *RMSE* of discrimination parameters under the MIRT model and the effort-moderated model were relatively stable, while those under the 3PL model were highly influenced by the independent variables and much larger. For the 3PL model, it can be seen that: (1) the *RMSE* of the 3PL model increased as the percentage of non-effortful simulees or the percentage of non-effortful responses within a non-effortful simulee increased. Besides, when the percentage of non-effortful responses reached 5%, different combinations of κ and π lead to different results, with the percentage of non-effortful responses within a non-effortful simulee showing a larger effect. (2) when the test was difficult, the *RMSE* of the standard 3PL model was much higher. The reason might be that the discrimination parameters in difficult tests were significantly underestimated by the 3PL model (see Table 1). (Wise and DeMars, 2006) study showed that the 3PL model yielded discrimination parameter estimates that were 0.25 higher on average than the effort-moderated model. One possible explanation is that, the probability of correctly responding to difficult items in difficult tests, predicted by the IRT model under effortful behaviors, may be equal to the probability of random guessing under non-effortful behaviors. Therefore, when data consisted of both effortful and non-effortful responses, the model was not able to differentiate examinees with various abilities, and the discrimination parameters would be underestimated comparing to those from data including only effortful responses.

Results for the difficulty parameters showed a different pattern. For one, the *RMSE* of difficulty parameters under the MIRT model and the effort-moderated model were stable as well. However, those under the 3PL model increased as the correlation between non-effortful responding and ability increased. For another, the use of all the models resulted in deteriorations in either easy tests or difficult tests, and the deteriorations were more evident and striking when a 3PL model was applied. As shown in Wise and DeMars (2006), different models tended to be in less agreement when items are easier, and there were virtually no differences between the models for the most difficult test. However, in the current study, different models resulted in poorer estimates of difficulty parameters and tended to be in less agreement in difficult tests as well. It may be attributed to the fact that the 3PL model showed positive biases of larger magnitude in difficult tests. For example, an examinee who may not answer a hard item correctly based on his/her true ability may give a right answer by guessing. In that case, the difficulty parameter would be underestimated using the original data.

For guessing parameters, Table 1 showed that both the *RMSE* and *BIAS* of the 3PL model were small, which meant that by fixing the prior distribution, the guessing parameter could be estimated accurately by the 3PL model.

**Figure 2:**
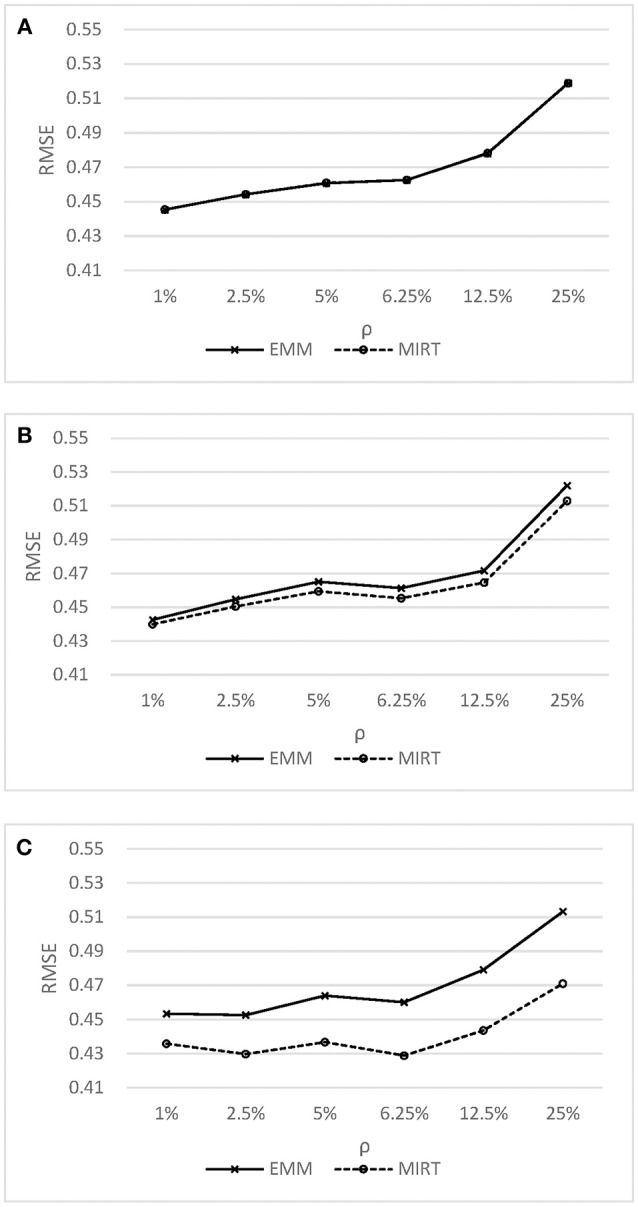
RMSE of ability estimates under the Effort Moderate Model MIRT model.**(A)** y = 0.0 **(B)** y = 0.4 **(C)** y = 0.8.

Figure 2 shows the *RMSE* of ability estimates under the MIRT model and the effort-moderated model. As shown in Figure 2, the MIRT model could obtain the ability estimates as accurate as or even better than the effort-moderated model. When the correlation between effortful responding and ability was high, the MIRT model was found to have lower *RMSE* compared to the effort-moderated model. This finding is expected, as previous research have shown that a latent modeling of the missing propensity may be effective in accounting for non-ignorable missing responses (Pohl et al., 2014; Rose et al., 2016). As in the test-taking non-effort context, the non-ignorable non-effortful responses were caused by the high correlation between non-effortful responding and ability (similar to the mechanism of missing not at random, MNAR). Therefore, comparing to the effort-moderated model, the MIRT model provided more accurate estimates in this condition.

Table 2 provides the *BIAS, RMSE*, and *Correlation* of ability estimates for different models. The impact of non-effortful responses on ability estimates based on original data can be summarized as follows: the 3PL model underestimated the ability parameters severely in most of the conditions, especially after the percentage of non-effortful responses reached 12.5%; a slightly higher *RMSE* was obtained when the correlation between non-effortful responding and ability was low; the ability estimates showed deteriorations in easy tests. The results are aligned with the findings in Rios et al. study (Rios et al., 2017). It was found that the difference between the probability of a correct response based on non-effortful and effortful behavior became larger as the test became easier, while the solution behavior resulted in much more accurate responses. For example, if examinee *i* with ability level of 0 was chosen from the sample, while three items (*j* = 1, 2, 3) with *a*_*j*_ = 1, *c*_*j*_ = 0.25 were chosen from the easy test (*b*_1_ = −1), moderated test (*b*_2_ = 0), and the hard test (*b*_3_ = 1) respectively. The probabilities of answering these three items correctly based on effortful behavior and non-effortful behavior were 0.798 and 0.250, 0.625 and 0.250, 0.452, and 0.250, respectively. It was obvious that the difference between the probabilities of two distinct behaviors was the greatest for the items from easy tests. As a result, when non-effortful responding manifests in easy tests, the impact of non-effortful responses on ability estimates would be significant. The *correlation* followed the same pattern as the *RMSE*.

**Table 2:** BIAS, RMSE, and correlation for ability estimates in study I.

**Condition**	**Level**	**κ**	**π**	***BIAS***			***RMSE***			***Correlation***		
				**3PL**	**Moderated**	**MIRT**	**3PL**	**Moderated**	**MIRT**	**3PL**	**Moderated**	**MIRT**
ρ	1%	10%	10%	0.045	0.006	0.009	0.458	0.447	0.440	0.890	0.893	0.896
	2.50%	10%	25%	0.141	0.002	0.008	0.557	0.456	0.448	0.861	0.887	0.892
		25%	10%	0.138	0.004	0.008	0.500	0.451	0.442	0.884	0.893	0.898
	5%	10%	50%	0.332	−0.006	0.010	0.905	0.472	0.458	0.797	0.883	0.890
		50%	10%	0.262	0.002	0.005	0.568	0.455	0.446	0.873	0.890	0.894
	6.25%	25%	25%	0.366	−0.003	0.007	0.765	0.461	0.449	0.833	0.886	0.892
	12.50%	25%	50%	0.792	−0.014	0.010	1.505	0.484	0.466	0.717	0.873	0.883
		50%	25%	0.706	−0.003	0.007	1.054	0.469	0.458	0.795	0.879	0.885
	25%	50%	50%	1.736	−0.022	0.007	2.473	0.518	0.501	0.661	0.860	0.870
γ	0.0			0.501	0.007	0.006	0.997	0.468	0.468	0.760	0.886	0.886
	0.4			0.504	−0.004	0.008	0.976	0.468	0.462	0.823	0.884	0.887
	0.8			0.501	−0.015	0.010	0.955	0.469	0.440	0.873	0.880	0.895
β	−1			0.523	−0.003	0.008	1.053	0.463	0.453	0.808	0.886	0.891
	0			0.477	−0.004	0.007	0.941	0.451	0.441	0.834	0.892	0.897
	1			0.506	−0.005	0.009	0.935	0.491	0.476	0.829	0.870	0.879

## Study II

### Design

In the second part of the simulation, datasets were generated based on the MIRT model [see Equations (3)(4)(5)] for 60 MC items, with *N* = 2,000. The distributions for generating item parameters and ability parameters were the same as in Study I. The ability and effortful propensity follow a bivariate normal distribution, with the correlation between them *cor*(θ_*i*_, ξ_*i*_) = γ. First, for 1 ≤ *k* ≤ *J*, the response data without non-effort Y^*^ were generated based on the 3PL model. Then, for *J*<*k* ≤ *2J*, the difficulty parameters for effortful propensity were drawn from a normal distribution $b_{k}\sim N\;\left({\bar{b}}_{k},1^{2}\right)$, where ${\bar{b}}_{k}$ varied across conditions. The effortful response indicators were generated similar to responses under IRT models. By manipulating different levels of ${\bar{b}}_{k}$ in the Rasch model, different levels of percentage of non-effortful responses could be generated. Finally, when the indicator *F*_*ik*_, for item *j* by person *i* was 0, the non-effortful response was generated as possessing a correct item response probability equal to chance level (0.25) to replace the response in Y^*^.

Three independent variables were considered: (1) the percentage of non-effortful responses in the sample (ρ), (2) the correlation between the effortful propensity and ability (γ), (3) test difficulty (β). The percentage of non-effortful responses had three levels: small (≈5%, ${\bar{b}}_{k}=$-3.5), moderate (≈12.5%, ${\bar{b}}_{k}$ = −2.5) and high (≈25%, ${\bar{b}}_{k}\;=$ −1.5). For the correlation, we set γ = 0.4 and γ = 0.8 to represent the conditions of non-ignorable non-effortful responses, and γ = 0 as the baseline to generate the ignorable non-effortful responses, mimicking the (missing completely at random) MCAR mechanism. Test difficulty had the same levels as in Study I: *b*_*j*_~ N (−1, 1^2^), *b*_*j*_~ N (0, 1^2^), and *b*_*j*_~ N (1, 1^2^).

The three independent variables and their corresponding levels were fully crossed, which resulted in a 3 × 3 × 3 design for a total of 27 conditions. One hundred replications were simulated for each condition. The models applied to the simulated dataset, the estimation process, and the evaluation criteria were the same as in Study I.

## Results

Tables 3, 4 summarize the results of Study II with respect to the item and person parameter estimates using the three models. Similar to Study I, *BIAS* and *RMSE* of the parameter estimates were substantially smaller under the MIRT model and the effort-moderated model than the 3PL model. For the MIRT model and the effort-moderated model, two trends were observed. One was that under the condition of ignorable non-effortful responses (γ = 0.0), the MIRT model performed as well as the effort-moderated model. The other was that the MIRT model could reduce the *BIAS* and *RMSE* when γ increased, especially when the percentage of non-effortful responses was high and the test was hard. As the conditions of ρ = 25% and γ = 0.8 in Study II were equivalent to the conditions of ρ = 25% and γ = 0.4 in Study I (where the correlation of the latent variables was about 0.4 under the MIRT model), the results of the two models under these conditions were examined. The differences of *RMSE* between the ability estimates under the two models were slightly larger in Study II than in Study I (Study I: 0.009, 0.008, and 0.011 for β = -1, 0, and 1; Study II: 0.014, 0.015, and 0.019 for β = -1, 0, and 1).

**Table 3:** BIAS and RMSE of item parameter estimates under different models in study II.

**Measures**	**Condition**	**Level**	***a***			***b***			***c***
			**3PL**	**Moderated**	**MIRT**	**3PL**	**Moderated**	**MIRT**	**3PL**
*BIAS*	ρ	5%	0.029	−0.009	−0.009	0.079	0.002	0.001	0.013
		10%	0.084	−0.010	−0.010	0.231	0.002	0.001	0.039
		25%	0.161	−0.014	−0.013	0.516	0.005	0.004	0.085
	γ	0	0.072	−0.011	−0.011	0.223	0.000	0.000	0.035
		0.4	0.097	−0.010	−0.010	0.286	0.001	0.001	0.048
		0.8	0.105	−0.012	−0.011	0.317	0.007	0.005	0.054
	β	−1	0.070	−0.009	−0.009	0.257	0.018	0.017	0.040
		0	0.088	−0.010	−0.010	0.285	0.003	0.002	0.045
		1	0.116	−0.013	−0.013	0.285	−0.013	−0.013	0.052
*RMSE*	ρ	5%	0.152	0.103	0.102	0.350	0.180	0.178	0.046
		10%	0.170	0.109	0.108	0.504	0.191	0.189	0.064
		25%	0.232	0.134	0.130	0.908	0.251	0.243	0.102
	γ	0	0.190	0.115	0.115	0.573	0.200	0.200	0.062
		0.4	0.181	0.113	0.112	0.584	0.205	0.204	0.073
		0.8	0.183	0.119	0.114	0.604	0.217	0.207	0.078
	β	−1	0.167	0.104	0.103	0.722	0.236	0.231	0.065
		0	0.170	0.107	0.105	0.574	0.169	0.165	0.072
		1	0.217	0.135	0.132	0.465	0.217	0.214	0.076

**Table 4:** BIAS, RMSE and correlation for ability estimates in study II.

**Condition**	**Level**	***BIAS***			***RMSE***			***Correlation***		
		**3PL**	**Moderated**	**MIRT**	**3PL**	**Moderated**	**MIRT**	**3PL**	**Moderated**	**MIRT**
ρ	1%	0.363	0.002	0.008	0.628	0.467	0.450	0.870	0.883	0.892
	5%	0.758	−0.006	0.007	0.989	0.482	0.458	0.835	0.875	0.888
	25%	1.690	−0.023	0.007	1.939	0.524	0.487	0.760	0.851	0.873
γ	0	0.934	0.006	0.006	1.213	0.489	0.489	0.745	0.874	0.874
	0.4	0.940	−0.009	0.007	1.189	0.491	0.480	0.820	0.873	0.879
	0.8	0.937	−0.024	0.008	1.154	0.494	0.427	0.890	0.864	0.900
β	−1	1.018	−0.006	0.008	1.260	0.479	0.456	0.814	0.877	0.889
	0	0.919	−0.010	0.006	1.170	0.483	0.459	0.830	0.875	0.888
	1	0.874	−0.011	0.007	1.126	0.511	0.481	0.837	0.859	0.876

### An Empirical Application

The real data set consisted of 1619 subjects' responses and response times on two tests: the matrix reasoning test and the analogical reasoning test. Each of the tests contained 30 MC items. Since the subjects were told that they could get feedbacks individually after scoring, it could be regarded as a high-stake setting.

Each scale was analyzed separately. First, non-effortful responses were flagged using a response time based the NT10 method. Ninety four subjects with total response time equal to 0 were removed. In addition, if a response time equal to or lower than 0 (mistaken record), it was recoded as missing. Afterwards, non-effortful responses were identified using the NT10 method, as this method was found to be effective for identifying non-effortful responses in a previous study (Wise and Ma, 2012). The three models used in the simulation study were fit to the data respectively. Moreover, as the matrix reasoning test consisted items with 8 options, while the analogical reasoning test consisted items with 4 options, the constant probability for non-effortful responses were fixed at 0.125 (1/8) and 0.25 (1/4) for them, respectively.

In general, the percentage of non-effortful responses was 4.8% for the matrix reasoning test and 0.3% for the analogical reasoning test. In the MIRT model, the effortful propensity could be obtained, which had a correlation of 0.06 with ability for the matrix reasoning test and 0.11 for the analogical reasoning test. The correlations were rather low as compared to our simulation conditions.

To assess the meaningfulness of the identified non-effortful responses and the performance of different models, the external validity was assessed. As both tests evaluated the reasoning ability of the students, the correlation of ability estimates of the two tests can be regarded as a measure for convergent validity. We hypothesized that in case the non-effortful responses couldn't reflect the real level of the latent trait of a student, the correlation between the ability estimates of these tests should be lower based on the 3PL model than the other two models. The results showed that, the correlation was 0.305 based on either the MIRT model or the effort-moderated model, and 0.272 based on the 3PL model. This implies that the test has less convergent validity under the 3PL model than that under the MIRT model or the effort-moderated model.

**Figure 3:**
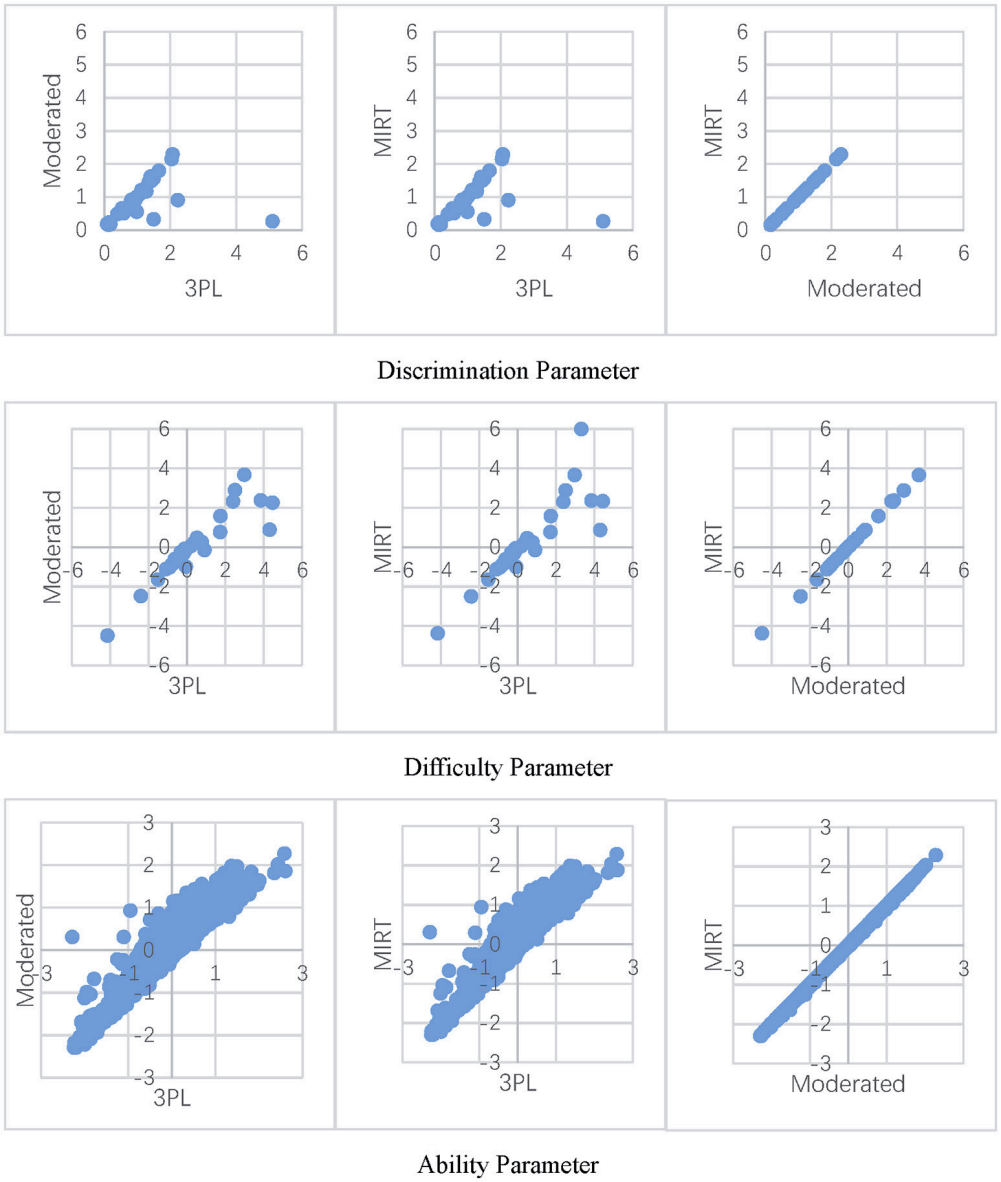
Parameter estimates under Different models.

Figure 3 shows the parameter estimates of the three models for the matrix reasoning test, which has a larger percentage of non-effortful responses. It presents that the MIRT model and effort-moderated model have very close parameter estimations. This result was consistent with those in the simulation study. Under a similar condition of ρ = 5% and γ = 0.0 in study II, the difference of the *RMSE* of the parameter estimates under the two models was <0.04. Moreover, in accordance with a previous study (Wise and DeMars, 2006), the 3PL model tended to overestimate the discrimination parameters compared to the other two models. Meanwhile, an interaction effect was apparent for the difficulty parameter. As the items got more difficult, the models tended to be in less agreement. For the ability parameter, if non-effort was neglected in the model, the examinee's proficiency was lower comparing to the models for non-effortful responses.

## Discussion

In low-stakes assessment situations, some examinees may be unmotivated and provide non-effortful responses. For the integrity of test results, stake-holders need to understand whether the non-effortful responding has a significant impact on parameter estimation in traditional measurement models, and if so, how to decrease this impact using advanced modeling approaches.

With two simulation studies, this article demonstrated that the impact of non-effortful responses on measurement outcomes depends on several factors: the percentage of non-effortful responses in the sample, the correlation between non-effortful responding and latent ability, and test difficulties. As expected, the *RMSE*s of item and ability estimates under the 3PL model increased as the percentage of non-effortful responses became larger, especially over 6.25%. Moreover, when the correlation between non-effortful responding and ability was low, a slightly higher *RMSE* was obtained for ability parameters, while the *RMSE* of the difficulty parameters decreased. The ability estimates showed deteriorations in easy tests, while the difficulty parameter had deteriorations in both easy and difficult tests.

Additionally, the MIRT model for non-effortful responses was evaluated, by comparing to the 3PL model and the effort-moderated model (Wise and DeMars, 2006) under various conditions. Unlike the existed models (e.g., the effort-moderated model), the propensity to answer effortfully can be estimated by the MIRT model as a continuous, latent dimension on its own. Consequently, not only the relationship of ability and effortful propensity can be investigated, but also this propensity can be saved for further studies, or added in a structural equation model to investigate the causes and dynamics of non-effortful behaviors. Furthermore, the MIRT model shows other desirable advantages as well. First, when non-effortful responding is present, even with a small amount (e.g., 1%), the MIRT model was found to provide accurate parameter estimates, similar to the effort-moderated model, which was shown to be more appropriate than unidimensional IRT models in the presence of non-effortful responses in previous studies (Wise and DeMars, 2006; DeMars and Wise, 2010). In the current study, even when responses were simulated based on the effort-moderated model, the MIRT model performed very well. Second, even if the non-effortful responses were ignorable (γ = 0.0), the over-fitting of the MIRT model didn't cause any issues. Third, when the correlation between non-effortful responding (or the effortful propensity) and latent ability was high, the non-effortful responses were non-ignorable, thereby the MIRT model could obtain more accurate results than the effort-moderated model. Fourth, the MIRT model is flexible. For one thing, it does not require assumptions about the patterns of test-takers' non-effortful responding. Therefore, it could be applied when test-takers behave in accordance with switching models, gradually decreasing effort models, or other potential models. For another, this model allows different multidimensional structures for both latent ability and the latent effortful propensity. Fifth, as the MIRT model is compatible with commonly used MIRT software, this method can be easily applied and widely used in practice. Sixth, the MIRT model can be generalized to deal with constructed respond (CR) items, as well as tests with mixed types of items. In contrast, the effort-moderated model can only deal with MC items. In the end, we applied the non-effort models to two sets of empirical data and had the following findings: (1) the convergent validity based on the MIRT model was similar to that based on the effort-moderated model, both of which were higher than that based on the 3PL model; (2) the MIRT model can obtain parameter estimates consistent with the effort-moderated model for this real data; (3) the MIRT model can provide estimates of the propensity of effortful responses and its relationship with ability simultaneously.

### Limitations and Future Research

One major limitation of this paper is that, the non-effortful responses were assumed to be accurately flagged in the simulation studies, as the focus of this study is to compare different models for non-effortful responses, not to identify non-effortful responses. However, such an assumption can often be violated in reality. Though a large number of approaches have been proposed for detecting non-effortful test-taking behavior (Wise and Ma, 2012; Guo et al., 2016), none of them can flag non-effortful behaviors exactly, and the selection of effort-detection method might affect the recovery of parameter estimates in IRT calibration. Furthermore, the application of MIRT methods has its own limitations. For instance, the MIRT model assumes a linear relationship between examinees' proficiency and their propensity to answer effortfully, which may be questioned in many applications. For example, a non-linear relationship between the two latent traits might exist because fewer test-takers at the low ability levels respond effortfully to an easy item than test-takers at the medium or high ability levels, whereas the proportion of test-takers of different ability levels responding effortfully to a difficult item might be similar. Hence the distribution of non-effortful responses could be multimodal due to different proficiency levels. That is to say, as an analogy to differential item functioning, a covariate (i.e., item difficulty) could exert an influence on effortful responding conditioning on ability, leading to differential non-effortful responses by examinees of the same proficiency. Under this situation, the MIRT model may obtain biased estimations of item and ability parameters.

The above-mentioned limitations suggest three potential areas of future research. The first is developing parametric methods for the identification of non-effortful responses. As noted above, despite numerous proposed methods of identifying non-effortful behaviors in both survey and cognitive assessments, many of the existed methods are nonparametric and difficult to replicate. This study has illustrated that the non-effortful responses can lead to biased parameter estimates, thus, it is important to figure out how to flag non-effortful responses accurately. A possible solution is to develop new parametric methods to identify non-effortful responses based on response times or other evidential cognitive sources, such as measures of eye-tracking.

Next, as the nature of examinees' effort and the mechanisms underlying the effort change during testing are still unknown (Debeer et al., 2014), the following studies could focus on the characteristics of items or examinees that may cause different levels of the propensity to answer effortfully. For example, well-designed questionnaires can be administered to explore what kind of covariates can moderate test-taking efforts. We believe these studies will shed light on test design as well as the improvement of examinees' test-taking effort.

Another aspect of future studies lies in developing new models to deal with the non-effortful responses. The IRTree model (De Boeck and Partchev, 2012; Debeer et al., 2017), which applies the logic of a tree-based model to the process of responding to an item, may provide an alternative way to model non-effortful responses. Regarding the definition of IRTree models, test-taking effort modeling has similar response process as the models require. For an examiner, the first process is to decide whether to take the full effort to answer the item. If the answer is yes, then the second process is to give his/her response based on the true ability. Similar to the MIRT model, the effortful tendency, the latent ability, and their correlations can be estimated simultaneously. Furthermore, under the framework of IRTree models, several hypotheses about the process underlying an item response can be proposed, as well as the related IRTree models. These IRTree models can be applied to real data for the hypothesis test and interpretation of the process.

## Author Contributions

YL: data analysis, paper writing; ZL: paper revision; HL: model design, paper revision; FL: paper revision.

### Conflict of Interest Statement

ZL was employed by company eMetric LLC, TX, USA. eMetric LLC provides support in the form of salaries for author ZL. The remaining authors declare that the research was conducted in the absence of any commercial or financial relationships that could be construed as a potential conflict of interest.
